# Physical Health Impairment and Exercise as Medicine in Severe Mental Disorders: A Narrative Review

**DOI:** 10.1186/s40798-022-00490-3

**Published:** 2022-09-15

**Authors:** Mathias Forsberg Brobakken, Mona Nygård, Eivind Wang

**Affiliations:** 1 Faculty of Health and Social Sciences, Molde University College, Molde, Norway; 2 Department of Psychosis and Rehabilitation, Psychiatry Clinic, St. Olavs University Hospital, Trondheim, Norway; 3 Department of Mental Health, Faculty of Medicine and Health Sciences, Norwegian University of Science and Technology (NTNU), 7491 Trondheim, Norway

**Keywords:** Severe mental illness, Psychiatry, Aerobic capacity, Maximal strength, 1RM, Power

## Abstract

**Background:**

Individuals with severe mental disorders (SMDs; schizophrenia spectrum disorders, bipolar disorder, and major depressive disorder) are not only suffering from their mental conditions; they also have an attenuated physical health, augmenting their overall critical condition.

**Objectives:**

We review and critically appraise the evidence based on (1) key physiological factors relating to aerobic endurance and skeletal muscle strength; (2) implications for physical function and health; and (3) effects of training interventions with different intensities evaluated in individuals with SMDs.

**Findings:**

Reductions in aerobic endurance factors, peak oxygen uptake (VO_2peak_) and walking work efficiency, are paralleled by reductions in maximal skeletal muscle strength and power. In turn, the poor aerobic endurance and muscle strength lead to impaired physical function, increased risk of lifestyle-related diseases, and ultimately early death. Exercise has the potential to counteract the attenuated physical health in people with SMDs. While aerobic endurance training is shown to increase VO_2peak_ due to plasticity of the oxygen transport system, strength training is documented to improve maximal muscle strength, power, and walking work efficiency as a result of adaptations in neuromuscular force developing factors.

**Conclusions:**

In conclusion, improvements in these key determinants for physical health appear to be achievable in people with SMDs despite many being challenged by motivational difficulties with attending regular exercise and have beneficial implications for physical function during activities of daily living, lifestyle-related diseases, and early death.

## Key Points

- Physical activity is often recommended in clinical treatment of people with severe mental disorders but appears not to result in increased life expectancy and reduced prevalence of CVD.
- Physical training is an effective countermeasure to improve the low aerobic endurance and skeletal muscle strength in these individuals, factors directly associated with CVD and all-cause mortality, and should thus be included in clinical practice.

## Introduction

People with severe mental disorders (SMDs; schizophrenia spectrum disorders, bipolar disorder, and major depressive disorder) have an estimated prevalence of ~ 1 to 18% worldwide [1–4]. Strikingly, people suffering SMDs have 10–20 years reduced life expectancy. The leading causes of death are lifestyle-related illnesses, in particular cardiovascular disease (CVD) [5–7]. While antipsychotic side effects and other factors such as smoking may augment poor physical health [8, 9], physical inactivity is suggested to be the origin of CVD development [10–12]. As a consequence, physical activities have been implemented as a countermeasure in treatment at a wide range of clinics around the globe [11, 13–18]. Paradoxically, the life expectancy gap in individuals with SMDs has continued to increase over the past 20–30 years [7, 19, 20]. Lack of access to and provision of health care, with subgroups of individuals particularly difficult to reach (e.g., relating to severity of illness) [21, 22], in combination with metabolic side effects of second-generation antipsychotics [23] may to some extent accentuate the inability to bridge the life expectancy gap. However, it should be questioned if many of the applied physical activities have a sufficient overload to improve critical components of physical health. Aerobic endurance and skeletal muscle strength, respectively, are acknowledged as key factors to healthy aging and longevity [24, 25], and a purposively judicious appraisal of the status of these measures in people with SMDs, and how they may effectively and substantially be improved by training interventions, is warranted.

The present article disseminates and critically appraises the existing literature on (1) aerobic endurance (maximal/peak oxygen uptake [${\dot{\text{V}}}{\text{O}}_{{2{\text{max}}}}$/${\dot{\text{V}}}{\text{O}}_{{2{\text{peak}}}}$] and walking work efficiency) and skeletal muscle strength factors (maximal strength [one-repetition maximum, 1RM] and power) in individuals with SMDs; (2) the implications for physical function and longevity; and (3) the beneficial impact of exercise at different intensities along with safety and feasibility considerations. Specifically, the article separates between the distinctly different physiological effects of aerobic endurance and strength training and their critical importance in the war against inactivity in this patient population.

## Search Criteria and Study Selection

The original articles reviewed were selected from a literature search on the PubMed/Medline database from inception to February 1, 2022. For the purpose of identifying studies reporting data on aerobic endurance factors, skeletal muscle strength, and physical function, searches for each SMD group were applied with the following combination of medical subject headings (MeSH) and specific terms: (exercise test OR aerobic capacity OR cardiorespiratory fitness, OR muscle strength OR maximal strength OR one-repetition maximum OR force-generating capacity OR power OR rate/rapid force development OR physical functional performance OR activities of daily living); AND (schizophrenia spectrum and other psychotic disorders OR bipolar and related disorders OR depressive disorder, major). To identify exercise interventions, searches for each SMD group were applied with the following combination of MeSH terms: (exercise OR endurance training OR high-intensity interval training OR resistance training); AND (schizophrenia spectrum and other psychotic disorders OR bipolar and related disorders OR depressive disorder, major). The literature search was performed individually by authors MFB and MN with relevant studies collated before inclusion and critical appraisal. Inclusion criteria were ≥ 1 of the target outcomes; endurance training applying an intensity of ≥ 70% of peak/maximal heart rate or a corresponding workload; or strength training with a load of ≥ 60% of 1RM, to ensure appropriate classification of protocols as endurance or strength training [26, 27]. Disagreements regarding the inclusion of studies were discussed between all authors until consensus was reached. Reference lists of reviews and included original articles were also searched manually to identify other relevant articles not previously found through electronic searches.

## Oxygen Uptake

### Physical Performance and Health: The Role of Maximal Oxygen Uptake

${\dot{\text{V}}}{\text{O}}_{{2{\text{max}}}}$, commonly recognized as the single most important factor for endurance performance [28, 29], is defined as the “greatest rate of oxygen utilization under any given set of conditions” [30] and reflects all factors in the oxygen transport chain, from air to mitochondria [31]. In clinical populations, it is relatively common that criteria for appropriate ${\dot{\text{V}}}{\text{O}}_{{2{\text{max}}}}$ determination [32] are not reached because many individuals experience difficulty exercising to volitional exhaustion due to motivation, symptoms, medication, or fear. In this case, the highest achieved oxygen uptake measured during a given test is referred to as ${\dot{\text{V}}}{\text{O}}_{{2{\text{peak}}}}$ [33]. Secondary testing criteria, such as respiratory exchange ratio, maximal/peak heart rate, and rating of perceived effort, are often applied to counter this shortcoming. Not being very faithful to the venerable Fick principle [34], which unmistakably states that both central and peripheral factors in the oxygen transport may limit the greatest oxygen utilization rate, the term cardiorespiratory (cardio- respiratory-) fitness is often used interchangeably with ${\dot{\text{V}}}{\text{O}}_{{2{\text{max}}}}$ in the clinical literature. Importantly, low ${\dot{\text{V}}}{\text{O}}_{{2{\text{peak}}}}$ is also well acknowledged to be an important risk factor for CVD and premature mortality. This may be unsurprising considering ${\dot{\text{V}}}{\text{O}}_{{2{\text{peak}}}}$ reflects cardiac function and output as well as all further steps in the oxygen transport chain to the mitochondria. Specifically, a ${\dot{\text{V}}}{\text{O}}_{{2{\text{peak}}}}$ reduction by 1 metabolic equivalent of task (MET; 3.5 ml·kg^-1^·min^-1^ of oxygen uptake) is associated with increased risk of all-cause mortality and CVD by 12–13% and 15%, respectively [24, 35]. The survival benefit per MET increase has been firmly established through a wide range of studies of symptomatic and asymptomatic men and women at different ages [36], and particularly poor prognosis seems to be present for individuals with the lowest values [24, 35]. Additionally, ${\dot{\text{V}}}{\text{O}}_{{2{\text{peak}}}}$ has also been shown to decrease by approximately 1 MET (~ 10%) per decade from the fourth decade of life [37].

### Peak Oxygen Uptake and Severe Mental Disorders

Although persons with SMDs have different mental conditions, all subpopulations appear to have a substantially reduced ${\dot{\text{V}}}{\text{O}}_{{2{\text{peak}}}}$ compared to healthy reference values [37]. In people with schizophrenia [38, 39], ${\dot{\text{VO}}}_{{2{\text{peak}}}}$ reductions typically range from − 7 to − 14 ml kg^−1^ min^−1^, while people with major depressive [40–43] and bipolar disorder [44, 45] display reductions of − 5 to − 10 and − 4 to − 5 ml kg^−1^ min^−1^, respectively, compared to reference populations (Table 1). Due to the application of direct pulmonary measurements during incremental exercise protocols to exhaustion, the gold standard for clinical ${\dot{\text{V}}}{\text{O}}_{{2{\text{peak}}}}$ assessment, a robust body of evidence supports reduced ${\dot{\text{V}}}{\text{O}}_{{2{\text{peak}}}}$ in people with SMDs. Interestingly, the extent to which the physiological age of people with SMDs is reduced, as expressed by ${\dot{\text{V}}}{\text{O}}_{{2{\text{peak}}}}$, also appears to mirror their 10–20 years of shorter life expectancy. However, identification of sex-specific ${\dot{\text{V}}}{\text{O}}_{{2{\text{peak}}}}$ values appears to be rare, particularly in people with major depressive and bipolar disorder. In some cases, only ${\dot{\text{V}}}{\text{O}}_{{2{\text{peak}}}}$ values relative to bodyweight (ml kg^-1^ min^−1^) are reported, which increases the difficulty of examining interstudy differences as bodyweight may differ considerably. Notably, ${\dot{\text{V}}}{\text{O}}_{{2{\text{peak}}}}$ is commonly tested on cycle ergometers due to safety concerns. This may result in 5–10% lower values compared to treadmill tests and should be taken into account when interpreting results across studies [46].

**Table 1:** Pulmonary measured peak/maximal oxygen uptake in people with severe mental disorders

Study	Population	${\dot{\text{V}}}{\text{O}}_{{2{\text{peak/max}}}}$ L min^−1^ (mL kg^−1^ min^−1^)						Men/women, *n*		Age, years		Protocol
		SMD		Healthy normative values		Difference in absolute values [and %]		SMD	Healthy references	SMD	Healthy references	
		Men	Women	Men	Women	Men	Women					
Brobakken et al. [38]	Schizophrenia spectrum disorders	3.13 ± 0.54 (34.5 ± 8.7)	2.18 ± 0.48 (26.4 ± 7.0)	4.20 ± 0.60 (47.4 ± 6.8)	2.50 ± 0.41 (37.9 ± 5.2)	− 1.07 [− 25%] (− 12.9) [− 27%]	− 0.32 [13%] (− 11.5) [30%]	28/20	28/20	35 ± 11	35 ± 11	Case–control study with individualized incremental treadmill test to exhaustion
Andersen et al. [39]	Schizophrenia spectrum disorders	2.30 ± 0.80 (26.0 ± 8.0)	1.80 ± ± 0.40 (24.0 ± 8.0)	3.30 ± 0.70 (39.8 ± 9.0)	2.10 ± 0.50 (31.0 ± 7.0)	− 1.00 [30%] (− 13.8) [35%]	− 0.30 [14%] (− 7.0) [23%]	40/27	405/389	37 ± 13	46 ± 12	Patients compared with population-based sample with modified Balke treadmill protocol to exhaustion
Donath et al. [43]	Major depressive disorder	–	2.07 ± 0.27 (30.2 ± 6.3)	–	2.26 ± 0.33 (35.8 ± 7.3)	–	− 0.19 [8%] (− 5.6) [15%]	0/15	0/15	38 ± 12	38 ± 12	Case–control study with standardized incremental cycle ergometer test to exhaustion
Boettger et al. [42]	Major depressive disorder	(34.0)		(40.0)		(− 6.0) [15%]		7/15	7/15	37 ± 13	37 ± 12	Case–control study with standardized incremental cycle ergometer test to exhaustion
Minghetti et al. [40]	Major depressive disorder	2.10 ± 0.50 (31.0 ± 7.2)		2.85 ± 0.49 (39.7 ± 8.0)^a^		− 0.75 [26%] (− 8.7) [22%]		14/46	73/63	36 ± 11	35 ± 3	Exercise intervention, standardized incremental cycle ergometer test to exhaustion
Kerling et al. [41]	Major depressive disorder	2.35 ± 0.67 (28.8 ± 8.2)		3.09 ± 0.55 (39.0 ± 8.0)^a^		− 0.74 [24%] (− 10.1) [26%]		26/16	91/86	42 ± 10	45 ± 3	Exercise intervention, standardized incremental cycle ergometer test to exhaustion
Schuch et al. [44]	Bipolar disorder	1.85 ± 0.32 (23.8 ± 4.1)		2.10 ± 0.43 (28.7 ± 5.5)		− 0.25 [12%] (− 4.9) [17%]		4/10	7/9	36 ± 9	35 ± 8	Patients compared with healthy with standardized incremental cycle ergometer test to exhaustion
Vancampfort et al. [45]	Bipolar disorder	(26.0 ± 7.3)		(30.4 ± 6.5)		(− 4.4) [14%]		6/14	6/14	48 ± 8	48 ± 8	Case–control study with standardized incremental cycle ergometer test to exhaustion

Data are mean and standard deviation if reported, and presented as sex-specific values if available, or sexes combined when not

${\dot{\text{V}}}{\text{O}}_{{2{\text{max}}}}$, peak/maximal oxygen uptake; SMD, severe mental disorders

^a^Age- and sex-specific healthy normative values; Edvardsen et al. [37]

While ${\dot{\text{V}}}{\text{O}}_{{2{\text{peak}}}}$ remains a strong predictor of mortality, the relationship between physical activity and ${\dot{\text{V}}}{\text{O}}_{{2{\text{peak}}}}$ is shown to be relatively weak and inconsistent in both people with schizophrenia spectrum disorders [38] and healthy adults [47], indicating that simply increasing physical activity without a concomitant increase in ${\dot{\text{V}}}{\text{O}}_{{2{\text{peak}}}}$ may not necessarily improve CVD and mortality risk prognosis.

### Indirect Estimation of Peak Oxygen Uptake

Because direct measurements require expensive equipment, experienced test personnel, and may be difficult to undertake for some individuals, ${\dot{\text{V}}}{\text{O}}_{{2{\text{peak}}}}$ is often estimated by indirect methods [48]. Unfortunately, it may result in gross inaccuracy [36]. Even when tests to exhaustion are applied, such as the Bruce [49] or BSU/Bruce ramp [50] protocols, the standard error of estimate is shown to be 3.2 and 3.4 ml kg^−1^ min^−1^, respectively. This implies that in 95 out of 100 cases, a patient with a ${\dot{\text{V}}}{\text{O}}_{{2{\text{peak}}}}$ of, for example, 35 ml kg^−1^ min^−1^ will display a value within a range of ~ 13 ml kg^−1^ min^−1^, which corresponds to ~ 50 to 60% difference in risk assessment of all-cause mortality and CVD. It may also camouflage the gap between populations and the true change in ${\dot{\text{V}}}{\text{O}}_{{2{\text{peak}}}}$ following endurance training interventions. Consequently, while indirect ${\dot{\text{V}}}{\text{O}}_{{2{\text{peak}}}}$ tests are certainly easier to carry out, results may be highly misleading.

### Walking Work Efficiency in People with Severe Mental Disorders

Another important factor for aerobic endurance performance [51], along with V̇O_2max_, is work efficiency, the ratio between work output and oxygen uptake [52, 53]. Physical work conducted less efficiently implies that human locomotion requires more energy and oxygen consumption, resulting in impaired aerobic endurance and poorer physical function during activities of daily living. In the clinical setting, it is, because of functional relevance during daily tasks, commonly assessed when walking. Walking work efficiency is shown to be 14% lower in people with schizophrenia spectrum disorders compared to healthy references [54]. Analyzing men and women separately, work efficiency was reduced by 17% in the men and tended to be reduced by 9% in the women. To the best of our knowledge, this is the only study that has assessed this functionally important factor for aerobic endurance in people with schizophrenia, and it appears that no studies have examined walking work efficiency in individuals with major depressive or bipolar disorder. However, reduced walking work efficiency by 12% has also been documented in people with substance use disorders, with no difference between sexes [55], as well as individuals with preexisting CVD [56, 57].

## Muscle Strength

### Physical Performance, Health, and Skeletal Muscle Strength

Maximal muscle strength and power are key components affecting functional performance in everyday life and have both been acknowledged as independent risk factors of all-cause mortality [58–60]. Maximal muscle strength is typically measured as the heaviest weight one can lift once in a given movement (1RM) and decreases by approximately 1% per year from the fourth decade of life [61]. Power is a sub-component of maximal muscle strength and a product of work over time, i.e., force × velocity [25]. Interestingly, power declines at a faster rate compared to maximal muscle strength, approximately 3% per year [62], and there is mounting evidence that of these two factors, power may be more strongly associated with functional performance with aging [63].

### Maximal Muscle Strength and Severe Mental Disorders

How maximal muscle strength is affected in individuals with SMDs is a growing area of research. Despite strong indications, there are still knowledge gaps in the literature in terms of whether, and to what extent, maximal muscle strength is reduced across all subpopulations of SMDs. Dynamic leg press 1RM is shown to be 13 and 19% lower in women and men with schizophrenia spectrum disorders, respectively, compared to age- and sex-matched controls (Table 2) [54]. Isometric unilateral hip and knee extension strength is shown to be 32 and 42% lower, respectively, in individuals with major depressive disorder compared to healthy controls [64]. While lower extremities maximal muscle strength appears to be undocumented in individuals with bipolar disorder, maximal isometric handgrip strength is shown to be consistently attenuated across all subpopulations with SMDs, with reductions ranging from 14 to 41% [65–67]. Apart from one study, our literature search failed to identify studies which included results describing sexes separately. Although relatively few studies have measured dynamic maximal muscle strength directly both in the lower and in the upper extremities in individuals with SMDs, the extent to which maximal strength is reduced, similarly to observations in ${\dot{\text{V}}}{\text{O}}_{{2{\text{peak}}}}$, seems to comply with the 10–20-year reduction in life expectancy.

**Table 2:** Maximal skeletal muscle strength and power in people with severe mental disorders

Study	Population	Maximal strength and power							Men/women, *n*			Age, years		Protocol
		SMD			Healthy normative values		Difference (%)		SMD	Healthy references		SMD	Healthy references	
		Men		Women	Men	Women	Men	Women						
Nygård et al. [54]	Schizophrenia spectrum disorders^a^	1RM: 14.2 ± 4.0 P: 22.0 ± 8.7		1RM: 10.6 ± 2.7 P: 14.5 ± 4.6	1RM: 17.5 ± 3.6 P: 31.3 ± 6.2	1RM: 12.2 ± 2.6 P: 19.3 ± 4.9	1RM: − 19% P: − 30%	1RM: − 13% P: − 25%	28/20	28/20		35 ± 11	35 ± 11	Case–control study with standardized incremental strength tests in leg press
Vancampfort et al. [65]	Schizophrenia spectrum disorders	Handgrip MVC: 41.7 ± 12.7 Abdominal muscle strength: 10 ± 8 Standing broad jump: 127 ± 50			Handgrip MVC: 48.6 ± 12.9 Abdominal muscle strength: 18 ± 7 Standing broad jump: 172 ± 32		Handgrip MVC: − 14% Abdominal muscle strength: − 46% Standing broad jump: − 26%		14/8	14/8		41 ± 10	40 ± 10	Case–control study with a standardized test battery of muscle strength and power
Bader et al. [64]	Major depressive disorder	Hip extension: 461.2 Knee extension: 245.2			Hip extension: 682.3 Knee extension: 421.0		Hip extension: − 32% Knee extension: − 42%		11/9	11/9		47	47	Case–control study with standardized isometric strength tests utilizing force cells
Lawrie et al. [67]	Major depressive disorder	Handgrip MVC: Right hand: 25.2 ± 10.8 Left hand: 20.0 ± 11.8			Right hand: 35.2 ± 8.4 Left hand: 33.9 ± 7.7		Right hand: − 28% Left hand: − 41%		8/12	4/11		44 ± 12	41 ± 13	Patients compared with healthy group with handgrip dynamometer performance averaged over three attempts
Vancampfort et al. [66]	Bipolar disorder	Handgrip MVC: 38.6 ± 11.4 Abdominal muscle strength: 12 ± 8 Standing broad jump: 135 ± 49			Handgrip MVC: 46.6 ± 12.4 Abdominal muscle strength: 18 ± 8 Standing broad jump: 168 ± 32		Handgrip MVC: − 17% Abdominal muscle strength: − 37% Standing broad jump: − 20%		16/14	16/14		41 ± 12	41 ± 11	Case–control study with a standardized test battery of muscle strength and power

Data are mean and standard deviation if reported, and presented as sex-specific values if available, or sexes combined when not

SMD, severe mental disorders; 1RM, one-repetition maximum (kg); P, power (N m s^−1^); MVC; maximum voluntary contraction (kg); abdominal muscle strength (repetitions per 30 s); standing broad jump (cm); hip/knee extension (N)

^a^Allometrically scaled values.

### Skeletal Muscle Power and Severe Mental Disorders

Skeletal muscle power in the lower extremities is shown to be impaired by 25% in men and 30% in women with schizophrenia spectrum disorders compared with healthy references (Table 2) [54]. Standing broad jump performance, a proxy for lower extremities power assessment, is also documented to be reduced by 26 and 20% in individuals with schizophrenia spectrum disorders [65] and bipolar disorder [66], respectively, supporting the finding of blunted ability to produce force rapidly in these individuals. Sex-specific results, again, also appear to be lacking for skeletal muscle power in people with major depressive and bipolar disorder, increasing the difficulty of firmly establishing if neuromuscular force production is equally impaired in both sexes. However, given that lower extremity maximal muscle strength and power appears substantially reduced also in patients with substance use disorders undergoing clinical treatment [68], it may be that attenuated neuromuscular function is a prominent feature across psychiatric groups.

### Testing of Maximal Muscle Strength and Power

To the best of our knowledge, data from dynamic tests of maximal muscle strength and power in the functionally important lower extremities are scarce, particularly pertaining to individuals with major depressive and bipolar disorder. Further, a range of tests have been utilized and maximal muscle strength is predominantly documented in upper extremities small muscle mass [65–67]. Measurements of lower extremities maximal muscle strength and power are relatively simple to conduct, and thus it is somewhat surprising that handgrip testing applying dynamometers appears to be a far more popular choice. However, the lower extremities ensure the ability to perform daily activities important for independent living, such as walking, stair climbing, and chair rising. Consequently, assessment of lower extremities muscle strength provides clinically relevant information. Additionally, since dynamic tests resemble human locomotion more closely than isometric measurements, the former will likely have higher transfer value to assessment of weight-bearing functional performance. As for V̇O_2_ measurements, direct assessment maximal muscle strength and power is preferable with regards to accuracy and reliability.

### The Importance of Allometric Scaling in Maximal Muscle Strength and Power Testing

A muscle’s force development potential is directly proportional to its cross-sectional area [69]. Maximal muscle strength and power are thus greatly affected by size of body proportions. People with SMDs are often overweight compared to healthy individuals, and direct comparisons of results between groups may be misleading. Comparing two geometrically similar individuals with different height and body mass, one would expect the larger individual to perform better in absolute terms of weight lifted (kg) for a given movement, while dividing force produced (cross-sectional area; *L*^2^) by body mass (volume; *L*^3^) would lead to underestimation of the performance. Allometric scaling accounts for this effect by dividing the outcome variable by body mass raised to the power of 0.67 (or *L*^2/3^) [69]. Previous studies have sought to include persons with SMDs and healthy references with similar characteristics by matching for age and sex [67], body mass index [65, 66], or height and weight [64]. This certainly leads to more accurate assessments; however, the application of allometric scaling may further facilitate intergroup/interstudy comparisons when different test protocols are utilized.

## Implications for Physical Function, CVD Risk, and Early Death

**Fig. 1:**
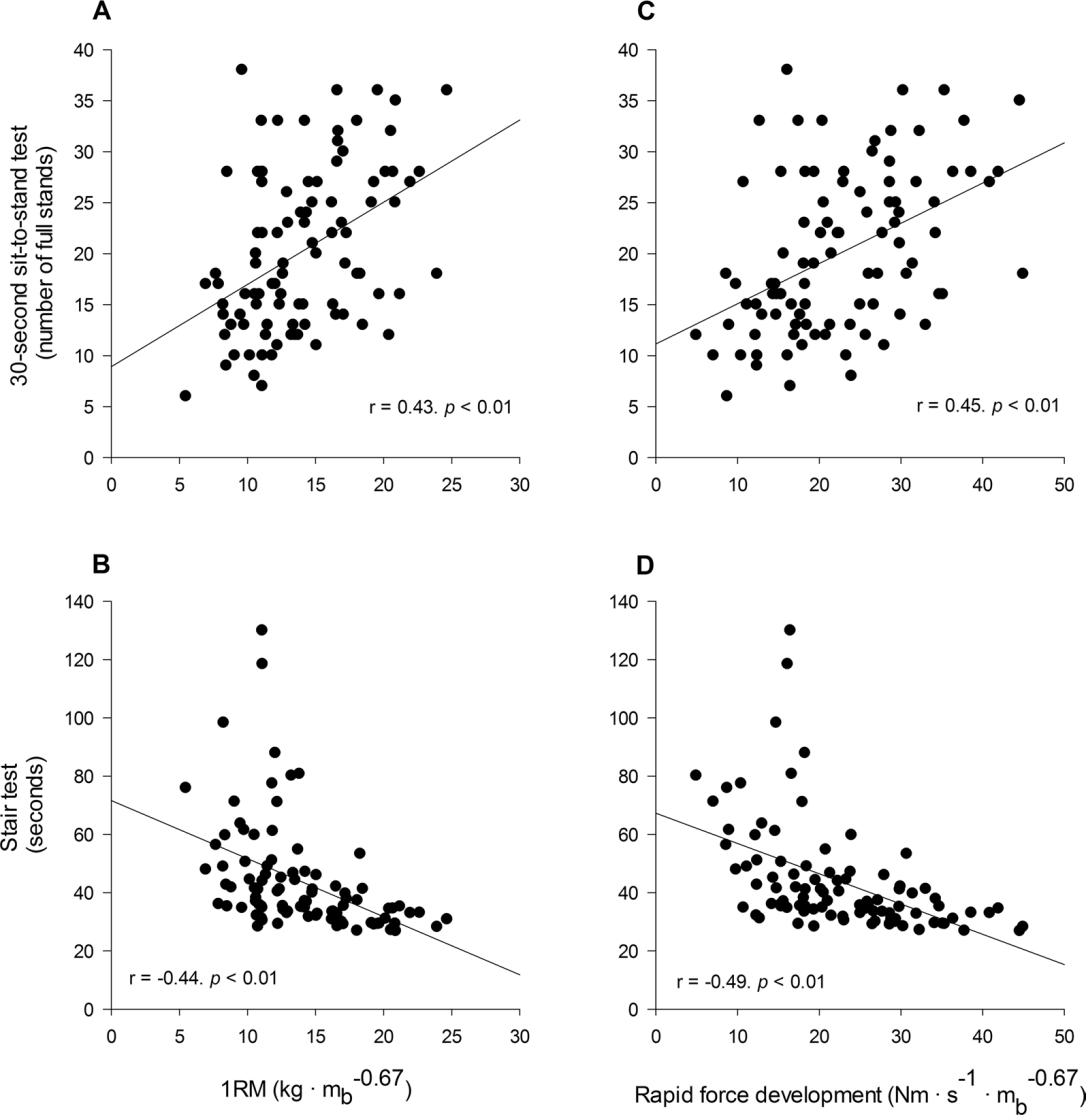
Relationship between muscular force-generating capacity and functional performance in force-demanding tasks. Associations between allometrically scaled one-repetition maximum (1RM) and performance in 30-s sit-to-stand test (**A**) and stair test (**B**). Associations between allometrically scaled rapid force development and performance in 30-s sit-to-stand test (**C**) and stair test (**D**). Used with permission from Nygård et al. [54].

Impairments in aerobic endurance and/or muscle strength imply that typical activities of daily living either require a higher percentage of an individual’s physical capacity, and thus are experienced as more vigorous, or not successfully completed at all. Two recent studies show that people with schizophrenia perform on par with 20–30 years older healthy references (− 20 to − 60%) during functional performance tests, i.e., walking, chair-rising, stair climbing, and balance [54, 70]. Walking performance is also shown to be reduced by − 23 and − 16% in individuals with major depressive [64] and bipolar disorder [71], respectively, compared to healthy controls. Notably, maximal muscle strength and power are associated (*r *= 0.4–0.7) with functional performance across all subpopulations (Fig. 1, schizophrenia spectrum disorders) [54, 64, 72]. Further, given robust evidence highlighting the inverse relationship between ${\dot{\text{V}}}{\text{O}}_{{2{\text{peak}}}}$ and maximal muscle strength with risk of CVD and mortality, the current literature underpins that these factors combine to attenuate functional performance and elevate the risk of CVD and early death, ultimately compounding the critical condition of individuals with SMDs.

## Exercise is Medicine for People with Severe Mental Disorders

For exercise to produce a measurable training effect, it must involve stimuli greater than the one usually encountered through everyday life in order to create an overload [73]. Exercise, i.e., subjecting a person to a training load or physical work, must be of adequate frequency, duration, and intensity to improve the function of key physiological systems [73]. By extension, when physical activity or exercise is available as treatment, a lack of overload due to one or more of these factors, which closely relates to changes in physical capacity and thus prognosis of adverse outcomes, may be of critical concern. Endurance and strength training represent two distinctly different types of training where the former has the purpose of overloading factors involved in oxygen transport and/or anaerobic energy production and the latter has the purpose of overloading factors involved in skeletal muscle force production. Both training types may independently contribute to adaptations that improve health and physical function in people with SMDs. Thus, it is important to consider the effects of endurance and strength training separately, and not combine them in the inaccurate term “physical activity.”

In individuals with SMDs, mean attrition rates of 17–27% have been reported from exercise interventions [16, 74, 75], on par or somewhat higher compared with control conditions and similar to what is commonly observed in sedentary individuals (18%) [76]. Importantly, for individuals who complete these interventions, attendance rates of 75–90% are usually reported with 2–3 training sessions per week (Tables 3, 4) [13, 41, 77–79]. This should be considered relatively high, albeit illness severity and the presence of supervision from qualified personnel are shown to influence dropout and exercise adherence [16, 74]. Exact dropout and attendance rates for individuals with bipolar disorder are unclear due to a dearth of exercise interventions specifically targeting this patient group. With that caveat, when exercise is available as treatment, the data suggest that exercise interventions are generally well accepted and feasible in individuals with SMDs.

**Table 3:** Effects of endurance training in people with severe mental disorders

Study	Population	Design and study duration	Participants^a^		Modality	Frequency and duration	Intensity	Results
			Included, *n*	Completers, *n* (%)				
Lin et al. [80]	Schizophrenia spectrum disorders	RCT, 12 weeks	40	29 (73%)	Walking and cycling	3 times/week 45–60 min	60–75% of HR_peak_	Directly measured treadmill ${\dot{\text{V}}}{\text{O}}_{{2{\text{max}}}}$ increased by 2.1 mL kg^−1^ min^−1^ (7%)
Scheewe et al. [81]	Schizophrenia spectrum disorders	RCT, 6 months	30	28 (93%)	Treadmill, cycle ergometer, rowing, cross trainer	2 times/week 40 min	45–75% of HR reserve	No change in directly measured ${\dot{\text{V}}}{\text{O}}_{{2{\text{max}}}}$ on cycle ergometer
Kimhy et al. [79]	Schizophrenia spectrum disorders	RCT, 12 weeks	16	13 (81%)	Treadmill, cycle ergometer, elliptical machine, active-play video game	3 times/week 45 min	60 to ≥ 90% of HR_max_	Directly measured cycle ergometer ${\dot{\text{V}}}{\text{O}}_{{2{\text{peak}}}}$ increased by 3.1 ± 3.3 mL kg^−1^ min^−1^(15%)
Brobakken et al. [13]	Schizophrenia spectrum disorders	RCT, 12 weeks	25	16 (64%)	Treadmill walking/running	2 times/week 4 × 4-min intervals	90% of HR_peak_	Directly measured treadmill ${\dot{\text{V}}}{\text{O}}_{{2{\text{peak}}}}$ increased by 3.1 ± 3.7 mL kg^−1^ min^−1^ (10%)
Heggelund et al. [85]	Schizophrenia spectrum disorders	CT, 8 weeks	16	12 (75%)	Treadmill walking/running	3 times/week 4 × 4-min intervals	90% of HR_peak_	Directly measured treadmill ${\dot{\text{V}}}{\text{O}}_{{2{\text{peak}}}}$ increased by 4.2 mL kg^−1^ min^−1^ (12%) Walking work efficiency increased by 12%
Andersen et al. [87]	Schizophrenia spectrum disorders	RCT, 12 weeks	43	34 (79%)	Treadmill walking/running	2 times/week 4 × 4-min intervals	90% of HR_max_	Directly measured treadmill ${\dot{\text{V}}}{\text{O}}_{{2{\text{max}}}}$ increased by 2.4 ± 4.5 mL kg^−1^ min^−1^ ^b^ (8%)
Kerling et al. [41]	Major depressive disorder	RCT, 6 weeks	22	22 (100%)	Cycle ergometer, treadmill, rowing, cross trainer, stepper, arm ergometry	3 times/week 45 min	50% of maximum workload	Directly measured cycle ergometer ${\dot{\text{V}}}{\text{O}}_{{2{\text{peak}}}}$ increased by 2.8 mL kg^−1^ min^−1^ (11%)
Danielsson et al. [77]	Major depressive disorder	RCT, 10 weeks	22	18 (82%)	Treadmill, cycle ergometer, cross trainer, stepper, rowing, jumping ropes	2 times/week 45 min	Intervals at 16–17 on the Borg scale	${\dot{\text{V}}}{\text{O}}_{{2{\text{max}}}}$ estimated to increase by 3.0 ± 0.5 mL kg^−1^ min^−1^ (13%) with submaximal cycle test
Romain et al. [17]	Psychosis patients	RCT, 6 months	38	21 (55%)	Treadmill walking/running	2 times/week 30 min, 30-s sprint intervals	80–90% of HR_max_	${\dot{\text{V}}}{\text{O}}_{{2{\text{max}}}}$ estimated to increase with submaximal treadmill test

Data are mean and standard deviation if reported

RCT, randomized controlled trial; CT, controlled trial; HR_peak_, peak heart rate; HR_max_, maximal heart rate; ${\dot{\text{V}}}{\text{O}}_{{2{\text{max}}}}$, maximal oxygen uptake; ${\dot{\text{V}}}{\text{O}}_{{2{\text{peak}}}}$, peak oxygen uptake

^a^Participants randomized/included in exercise training groups reported

^b^Increase only in participants instructed by competent personnel.

**Table 4:** Effects of strength training in people with severe mental disorders

Study	Population	Design and study duration	Participants^a^		Modality	Frequency and duration	Intensity	Results
			Included, *n*	Completers, *n* (%)				
Leone et al. [88]	Schizophrenia spectrum disorders	CT, 8 weeks	8	8 (100%)	Seven lower and upper extremity exercises with weight apparatus	2 times/week 4 weeks of 3 × 6 repetitions 4 weeks of 3 × 25 repetitions	80% of 1RM 50% of 1RM	1RM increase in all exercises, including leg and bench press by 25 (25%) and 16 kg (25%), respectively
Silva et al. [89]	Schizophrenia spectrum disorders	RCT, 20 weeks	14	12 (86%)	Seven lower and upper extremity exercises with weight apparatus	2 times/week 6 weeks of 2 × 10–15 repetitions 14 weeks of 3 × 6–12 repetitions	40–70% of 1RM 75–85% of 1RM	Only chest press and arm extension 1RM increased by 30 (27%) and 46 kg (45%), respectively
Strassnig et al. [92]	Schizophrenia spectrum and bipolar disorder	CT, 8 weeks	23	12 (52%)	Eleven lower and upper extremity exercises with pneumatic strength apparatus	2 times/week 3 × 10–12 repetitions with high intended concentric velocity	% of 1RM not reported	1RM and power increased for all exercises, including leg extension (37 kg, 17%; power: 26%) and chest press (9 kg, 19%; power: 13%)
Heggelund et al. [94]	Schizophrenia spectrum disorders	CT, 8 weeks	7	6 (86%)	Leg press with weight apparatus	3 times/week 4 × 4 repetitions	90% of 1RM	Leg press 1RM increased by 83 kg (38%) Walking work efficiency increased by 20%
Nygård et al. [95]ara>	Schizophrenia spectrum disorders	RCT, 12 weeks	25	17 (68%)	Leg press with weight apparatus	2 times/week 4 × 4 repetitions	90% of 1RM	Leg press 1RM and power increased by 51 kg (29%) and 43 N m s^−1^ (20%), respectively
Moraes et al. [90]	Major depressive disorder	RCT, 12 weeks	9	9 (100%)	Four lower and upper extremity exercises with weight apparatus	2 times/week 3 × 8–12 repetitions	70% of 1RM	Maximal strength (reported as total training load, not specified for each exercise modality) increased by ~40%
Leone et al. [78]	Mood disorders	CT, 8 weeks	7	7 (100%)	Seven lower and upper extremity exercises with weight apparatus	2 times/week 4 weeks of 3 × 6 repetitions 4 weeks of 3 × 25 repetitions	80% of 1RM 50% of 1RM	1RM increase in all exercises, including leg and bench press by 19 (25%) and 6 kg (13%), respectively

Data are presented as mean and% change

RCT, randomized controlled trial; CT, controlled trial; 1RM, one-repetition maximum

^a^Participants randomized/included in exercise training groups reported.

### Endurance Training and Severe Mental Disorders

#### Moderate Aerobic Intensity

Although ${\dot{\text{V}}}{\text{O}}_{{2{\text{peak}}}}$ is shown to be 1.5–3 METs lower in individuals with SMDs, there is promising evidence that endurance training may counteract the low values (Table 3). Applying pulmonary ${\dot{\text{V}}}{\text{O}}_{{2{\text{peak}}}}$ measurements, Lin et al. [80] demonstrated that 12 weeks of moderate intensity (60–75% of peak heart rate) endurance treadmill training for 45–60 min, three times a week, resulted in improvements of 2.1 ml kg^−1^ min^−1^ in people with schizophrenia spectrum disorders. In contrast, using indirect ${\dot{\text{V}}}{\text{O}}_{{2{\text{peak}}}}$ estimation, Scheewe et al. [81] reported no change in ${\dot{\text{V}}}{\text{O}}_{{2{\text{peak}}}}$ after 6 months of moderate intensity (45–75% of heart rate reserve) endurance training performed twice a week. The training intervention, however, may have counteracted a decline, as this was observed following control conditions. In individuals with major depressive disorder, 6 weeks of moderate intensity (50% of maximal workload) endurance training conducted for 45 min three times a week, improved pulmonary assessed ${\dot{\text{V}}}{\text{O}}_{{2{\text{peak}}}}$ by 2.8 ml kg^−1^ min^−1^ [41]. The relatively larger improvement may relate to the particularly low fitness observed in the subjects (27.1 and 26.5 ml kg^−1^ min^−1^). Interestingly, individuals with a very low ${\dot{\text{V}}}{\text{O}}_{{2{\text{peak}}}}$ (< ~30 ml kg^-1^ min^−1^) are primarily shown to be limited by working muscles’ ability to utilize oxygen during maximal effort, while this is suggested to not be the case for individuals with a somewhat higher ${\dot{\text{V}}}{\text{O}}_{{2{\text{peak}}}}$ [82]. Thus, a very low initial ${\dot{\text{V}}}{\text{O}}_{{2{\text{peak}}}}$ implies that individuals are more easily exposed to a training overload, have a larger potential for improvement, and that their training-induced adaptations may be governed by other physiological factors compared to less untrained counterparts.

#### High Aerobic Intensity

**Fig. 2:**
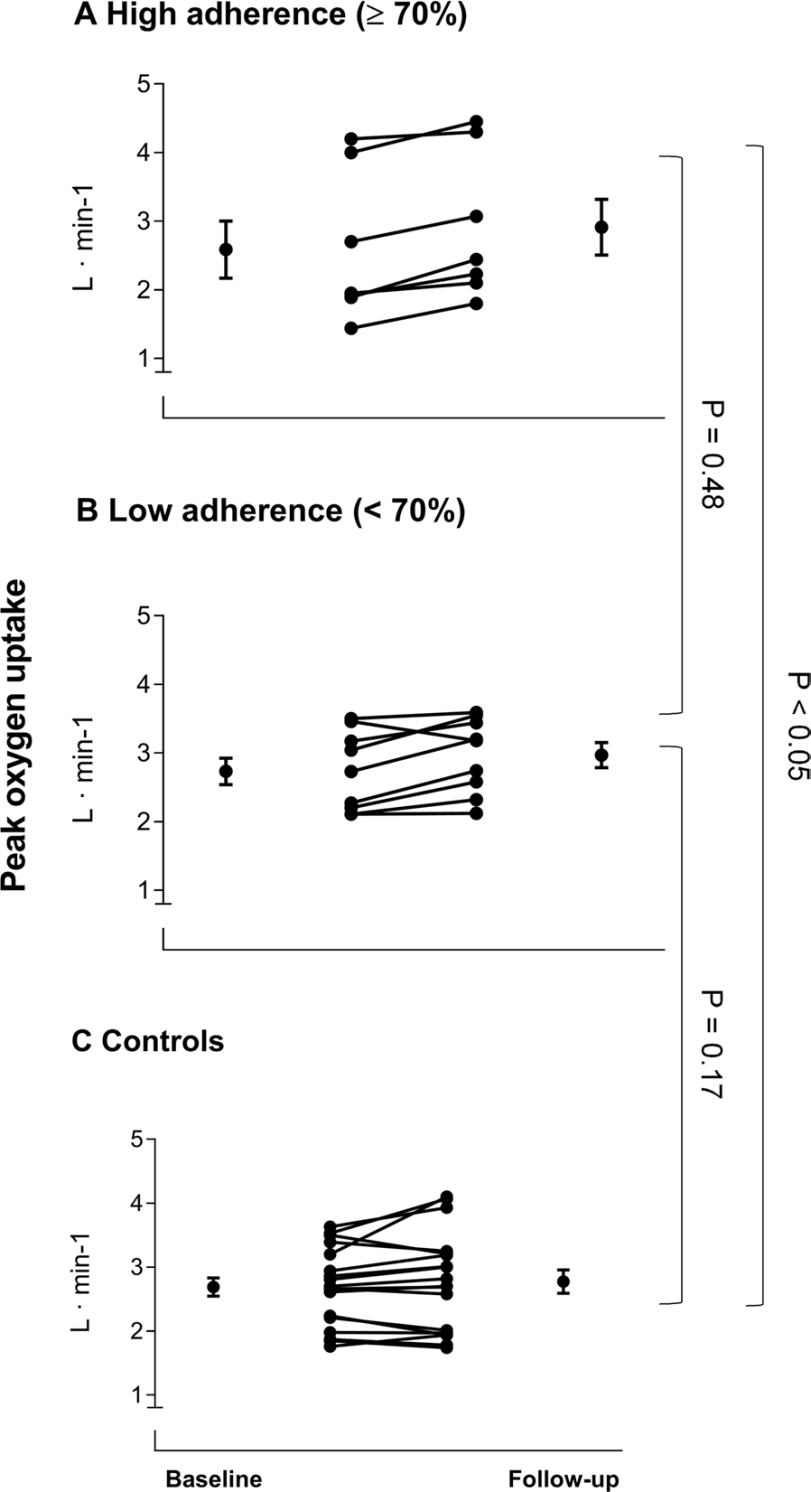
Individual values of peak oxygen uptake (l/min) pre- and post-12 weeks of aerobic interval training for the training groups with high adherence (**A**), low adherence (**B**) and controls (**C**). High adherence was set as completion of ≥ 70% of training sessions per protocol (*n* = 7) and low adherence as ≤ 70% of training sessions per protocol (*n *= 9). Values reported are mean and standard error. Used with permission from Brobakken et al. [13].

Similar to what has been observed in healthy individuals [53] and other patient populations [83, 84], endurance training with high aerobic intensity seems to yield greater increases in ${\dot{\text{V}}}{\text{O}}_{{2{\text{peak}}}}$ compared to endurance training with moderate aerobic intensity. In line with this notion, high-intensity aerobic interval training (4-min intervals at ~ 90% of peak heart rate with intermittent active rest periods) was demonstrated to increase pulmonary measured treadmill ${\dot{\text{V}}}{\text{O}}_{{2{\text{peak}}}}$ by 3.1–4.2 ml kg^−1^ min^−1^ (~ 1 to 1.5 METs) in men and women with schizophrenia spectrum disorders [13, 85]. This rapid improvement toward normality has been documented after 24 training sessions following 8–12 weeks in in-patients with schizophrenia undergoing residential treatment as well as out-patients (Fig. 2). Similarly, in the same SMD population, 12 weeks of training with moderate-to-high aerobic intensity (60 to ≥ 90% of peak heart rate) performed for 45 min, three times a week, increased ${\dot{\text{V}}}{\text{O}}_{{2{\text{peak}}}}$ by 3.1 ml kg^−1^ min^−1^ [79]. The high aerobic intensity-induced ${\dot{\text{V}}}{\text{O}}_{{2{\text{peak}}}}$ improvements appear to be on par with increases of ~1 to 1.5 METs observed in other cohorts, such as patients with substance use disorder [86], coronary artery disease [83], and young adults [53]. Although one study failed to document aerobic interval training-induced improvement in ${\dot{\text{V}}}{\text{O}}_{{2{\text{peak}}}}$ with intention-to-treat analyses in people with schizophrenia [87], explorative ad hoc analyses revealed a ${\dot{\text{V}}}{\text{O}}_{{2{\text{peak}}}}$ difference of 4.7 ml kg^−1^ min^−1^ after training between subjects instructed by personnel with (2.4 ml kg^−1^ min^−1^ increase) or without (2.2 ml kg^−1^ min^−1^ decrease) competence in supervising the sessions.

People with major depressive disorder also increased their ${\dot{\text{V}}}{\text{O}}_{{2{\text{peak}}}}$ with ~ 1 MET, as estimated with a submaximal cycle test, after 10 weeks of interval training (16–17 on the Borg scale) [77]. Our literature search did not identify any prospective endurance training interventions specifically targeting individuals with bipolar disorder. However, a study by Romain et al. [17] documented that treadmill intervals with 30-s exercise bouts improved indirectly estimated ${\dot{\text{V}}}{\text{O}}_{{2{\text{peak}}}}$ and was well accepted by a group of overweight individuals with psychosis in which bipolar disorder constituted 30% of the cohort.

Although high aerobic intensity training is typically applied to improve ${\dot{\text{V}}}{\text{O}}_{{2{\text{peak}}}}$ in people with lifestyle-related illnesses, it is also shown to improve walking work efficiency by 12% (19.8–22.2%) in people with schizophrenia spectrum disorders, yielding a substantial reduction in oxygen cost of submaximal walking. This improvement has previously been observed in healthy adults [53] and heart failure patients [84], with work efficiency improvements of 10 and 15%, respectively, being reported. Few studies have previously documented the effects of endurance training on work efficiency in other cohorts with SMDs. Minghetti et al. [40] reported that heart rate during a submaximal oxygen uptake test tended to decrease (4%) following 12 weeks of sprint interval training in men and women with major depressive disorder, but without a concomitant change in oxygen uptake, which may relate to the different exercise and testing conditions applied, or to the efficacy of the training protocol in improving walking work efficiency specifically.

Collectively, the data suggest that while people with SMDs display reduced aerobic endurance, they may benefit greatly from the effects of endurance training. Endurance training with moderate aerobic intensity evidently may lead to important ${\dot{\text{V}}}{\text{O}}_{{2{\text{peak}}}}$ increases in subjects with the lowest values; however, incorporating higher aerobic intensities appears to induce greater gains in ${\dot{\text{V}}}{\text{O}}_{{2{\text{peak}}}}$.

### Strength Training and Severe Mental Disorders

#### Moderate Intensity

The interest in offering individuals with SMDs strength training as part of clinical treatment is growing [11] and has promising results (Table 4). Most interventions have included people with schizophrenia spectrum disorders and moderate training intensities. Leone et al., including people with schizophrenia, showed that after 8 weeks of strength training at 50–80% of 1RM, leg and bench press 1RM increased by 25 (25%) and 16 kg (25%), respectively [88]. Moreover, Silva et al. [89] showed improvements in chest press (30 kg; 27%) and arm extension (46kg; 45%) 1RM in men with schizophrenia who partook in 20 weeks of multimodal lower and upper extremities strength training, twice weekly, at an intensity increasing gradually from 40 to 85% (2–3 sets of 6–15 repetitions). However, the subjects did not improve their performance in some of the other exercises involved in the program, indicating that the training load applied may have been too low or not well tolerated.

In older (60–80 years old) people with major depressive disorder, maximal strength increased by ~ 40% following 12 weeks of strength training twice weekly with moderate intensity (3 sets of 8–12 repetitions) in both lower and upper extremities exercises [90]. Although a direct comparison is challenging due to lack of absolute load per exercise being reported, this improvement appears to be on par with the 35% (24kg) increase in leg extension 1RM previously observed in young adults following conventional strength training with moderate intensity (3 sets of 10 repetitions) after 24 training sessions [91].

Our literature search failed to identify prospective strength training interventions targeting people with bipolar disorder specifically; thus, it is unclear if the results from other SMDs extend to this group. However, a pilot study of 7 individuals with mood disorders, predominantly consisting of bipolar disorder, showed increased 1RM by 19 (25%) and 6 kg (13%) in leg and bench press, respectively, after 8 weeks of strength training at 50–80% of 1RM performed to exhaustion [78]. This improvement was accompanied by 7 cm (37%) increased vertical jump performance. Further, in a mixed sample consisting of both people with schizophrenia spectrum disorders and bipolar disorder, Strassnig et al. [92] applied a moderate-intensity (3 sets of 10–12 repetitions) strength training protocol twice a week for 8 weeks, with emphasis on high intended velocity in the concentric phase, improving 1RM and power in exercises such as leg extension (1RM: 37 kg, 17%; power: 26%) and chest press (1RM: 9 kg, 19%; power: 13%).

#### High Intensity

**Fig. 3:**
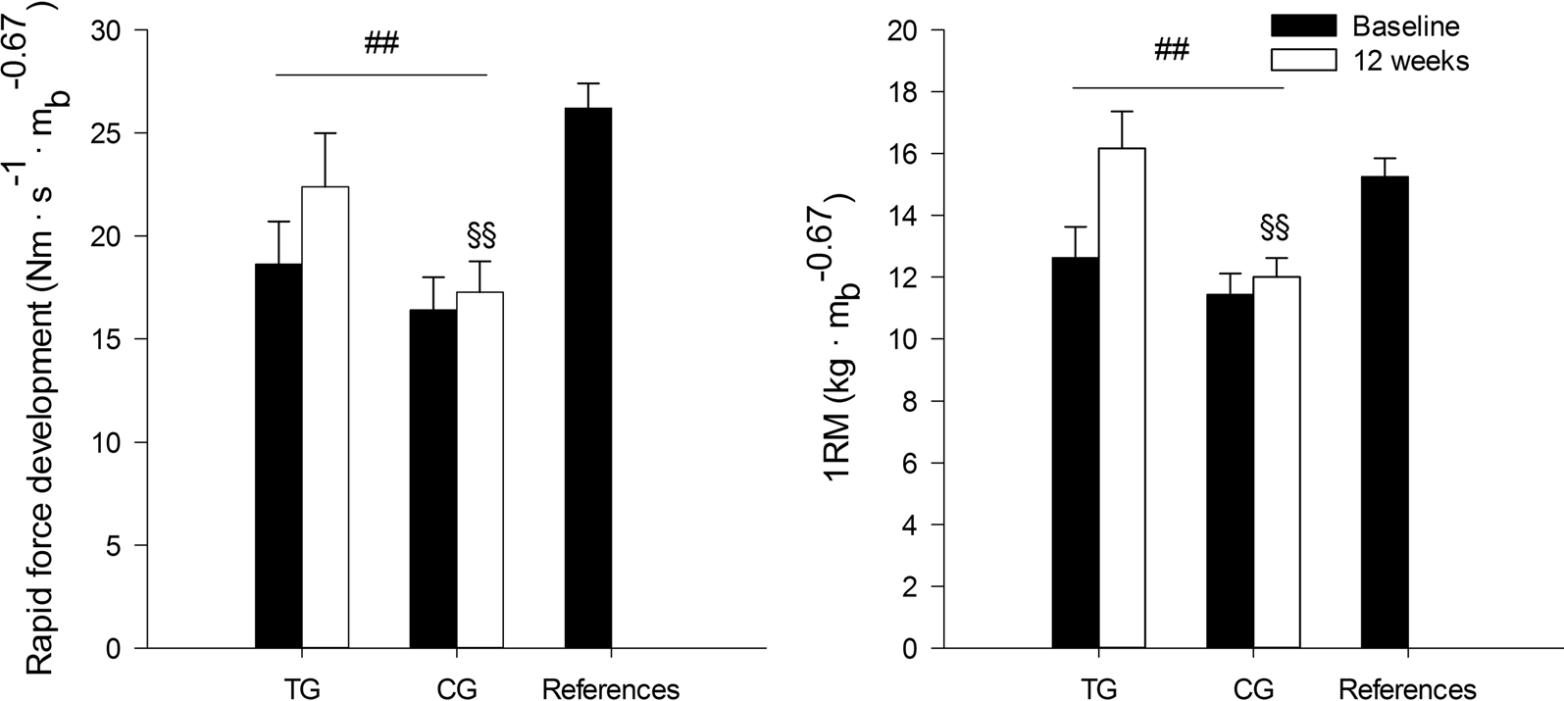
12-Week effects of maximal strength training on scaled rapid force development and one-repetition maximum (1RM) in the per-protocol training group (TG; *n *= 17) and control group (CG; *n *= 19) compared to healthy references. Patients included in the per-protocol analyses completed ≥ 50% of the training sessions. Values are mean and standard error. ^##^*P * <  .01 difference in change between groups, ^§§^*P *< .01 difference from healthy references at 12 weeks. Used with permission from Nygård et al. [95].

Two studies in people with schizophrenia spectrum disorders have applied strength training with high intensity (4 sets of 4 repetitions at 90% of 1RM), referred to as maximal strength training (MST) in the studies. MST aims to improve muscle strength predominantly through neural adaptations by applying heavy loads and maximal intended velocity. However, an MST-induced increase in fast twitch muscle fiber area and percentage has also been documented [93]. One of the MST studies revealed that leg press 1RM increased by 83 kg (38%) in in-patients with schizophrenia [94], while the other study revealed an increase in leg press 1RM by 51 kg (29%) and power by 43 N m s^−1^ (20%) in out-patients with schizophrenia spectrum disorders [95]. Notably, in the latter study, subjects who performed ≥ 50% of the training sessions demonstrated normalization of both 1RM and power compared to healthy references after 24 sessions (Fig. 3) [95]. Similar increases in 1RM and power following MST have also been observed in, for example, patients with substance use disorders [96, 97], coronary artery disease [56], peripheral arterial disease [57], and young adults [91]. However, a significant caveat may be that the increase in muscle strength following MST was inversely associated (*r *= − 0.5) with defined daily dose of antipsychotic medication [95], suggesting that subjects undertaking greater doses may experience blunted ability to improve muscle force production with strength training.

In addition to improving muscle strength, strength training, similarly to the improvement observed after endurance training, has also shown its potential to mitigate reduced walking work efficiency present in these individuals. In fact, MST is shown to increase walking work efficiency by 20% (17.3–20.7%) in men and women with schizophrenia spectrum disorders [94]. In contrast, another study in people with schizophrenia reported no change in walking work efficiency following MST [95], which may be due to greater variance in walking disabilities and energy expenditure, causing less improvement. The effects of strength training on work efficiency have, to our knowledge, not been replicated in other cohorts with SMDs. However, studies in people with lifestyle-related illnesses, such as coronary artery disease [56], peripheral arterial disease [57], and healthy young adults [91], have reported similar reductions in oxygen cost of walking after MST, likely due to a shift in the motor unit recruitment spectrum and contraction cycle phase [93]. Taken together, the evidence suggests that individuals with SMDs experience the same plasticity in the neuromuscular system and that performing high-intensity strength training may improve walking work efficiency.

Considering effect sizes, the same seems to apply for strength training as for endurance training; important improvements in 1RM and power are expected following training with moderate intensity in individuals with the poorest starting point. However, strength training which applies higher intensities appears to improve skeletal muscle strength more. The importance of high intensity is also highlighted in a current meta-review regarding exercise as treatment for individuals with SMDs [11]. If strength training is to be offered as part of treatment for people with SMDs, applying the most efficacious training regime in standard clinical practice should be considered.

### Safety Considerations

For endurance and strength training to be delivered as, or in conjunction with, standard clinical care, and act as effective countermeasures to poor physical health, the treatment must be considered safe. Notably, endurance and strength training, also with high intensities, are prescribed at a lower relative intensity than a subject’s maximal physical capacity. In individuals with low ${\dot{\text{V}}}{\text{O}}_{{2{\text{peak}}}}$, such as individuals with SMDs, this typically results in the treadmill endurance training being performed at uphill walking pace, generally considered to be a strenuous activity at high aerobic intensity but with low impact on the lower extremities [98]. For strength training, although the eccentric action phase may produce higher forces, maximal force is produced during the concentric phase, yet both lifting phases are performed in a slow and controlled manner due to heavy external loads, reducing risk of muscle injuries [93, 99]. Furthermore, given that these training regimes have been applied in other populations arguably frailer, such as heart patients [98], peripheral arterial disease patients [100, 101], old adults [93], hip fracture patients [102], and osteoporotic women [103], they should be considered safe also for individuals with SMDs [104].

### Endurance and Strength Training: Implications for Physical Function, CVD Risk, and Early Death

Increased aerobic endurance and/or skeletal muscle strength following training should result in improved physical function in individuals with SMDs. Indeed, a combination of high-intensity aerobic endurance training and MST did improve ${\dot{\text{V}}}{\text{O}}_{{2{\text{peak}}}}$, 1RM, and power along with chair-raising performance (14%) in people with schizophrenia spectrum disorders [13, 95]. Although the improvement in this functional performance test was strongly related (*r *= 0.6) to the increase in power [95], it was not apparent as an intergroup difference with no further improvements observed in walking or stair climbing performance. This is in contrast with what is typically observed in older adults [25], and indicates that the interplay between these factors may be influenced by illness-specific factors such as antipsychotics or sedation [105]. Two previous studies which applied aerobic endurance and strength training along with functional performance exercises in people with schizophrenia spectrum [88] and mood disorders [78] documented improved estimated ${\dot{\text{V}}}{\text{O}}_{{2{\text{peak}}}}$, 1RM, and power, as well as chair-raising (~ 20 to 25%) and stair climbing (~ 40%) performance. Although aerobic endurance and muscle strength are, in of themselves, measures of physical function, specific training as add-on treatment may be warranted for additional improvements in typical activities of daily living.

Given that ${\dot{\text{V}}}{\text{O}}_{{2{\text{peak}}}}$ and muscle strength are independently related to CVD and all-cause mortality, it is encouraging that physical training, particularly applying high intensities, elicits adaptations similar to previous observations in healthy adults and lifestyle-related illnesses. Notably, a 1 MET increase in ${\dot{\text{V}}}{\text{O}}_{{2{\text{peak}}}}$ as observed following endurance training is associated with reduced risk of all-cause mortality by 12% and CVD by 13–15% [24, 35]. Further, comparing terciles in maximal muscle strength, being in the middle–upper strength cohort is associated with 20–26% less risk of early death from all causes [58], underlining the importance of applying efficacious training regimes for individuals with SMDs who display reduced life expectancy from physical comorbidity.

## Conclusions and Perspectives

Recognizing the paradox that recommendations of physical activity in clinical treatment of people with SMDs do not result in increased life expectancy and reduced prevalence of CVD, a closer look at training interventions that have returned effective results is needed. In conclusion, the current review confirms that key physiological factors for health and longevity like ${\dot{\text{V}}}{\text{O}}_{{2{\text{peak}}}}$, walking work efficiency, maximal skeletal muscle strength and power, are reduced in individuals with SMDs. However, effective training interventions, ensuring sufficient overload on factors involved in oxygen transport and skeletal muscle force production, are demonstrated to counteract this reduction. Particularly, promising results appear to be evident after interventions incorporating higher training intensities. Compliance rates following such interventions are also high. Of notable concern, our literature search failed to identify prospective clinical trials with physical training specifically targeting people with bipolar disorder. Integrating effective training as part of standard clinical practice not only seems feasible and well accepted by people with SMDs yielding relatively low attrition, but may also improve physical function, cardiovascular health, and ultimately life expectancy.

Physical training is a well-acknowledged, feasible, and efficacious countermeasure to improve the low aerobic endurance and skeletal muscle strength observed in individuals with SMDs, factors directly associated with all-cause mortality. It is therefore concerning that it is rarely integrated, or delivered as adjunctive treatment, in standard clinical care for these patient groups. Variation in rates and causes for dropout from exercise interventions between diagnostic groups of SMDs suggest that a patient-centered tailored approach, optimally involving primary and secondary healthcare services [106] or strategies to promote autonomous motivation [107], may facilitate treatment adherence. This comprehensive support may be considered resource demanding; however, SMDs are associated with substantial societal cost due to the often-required extensive healthcare services from an early age. It is time to improve clinical practice to include effective physical training. Considering the increased mortality and poor somatic health of people with SMDs, it could be regarded unethical not to do so as it has the potential to improve key factors for health and longevity without detrimental side effects. Structured physical training may not only be beneficial for the individuals and their families but may also help reduce dependence on healthcare services.

## Data Availability

Data sharing is not applicable to this article as no datasets were generated or analyzed during the current study.
